# Retraction Note to: MicroRNA-330-3p promotes cell invasion and metastasis in non-small cell lung cancer through GRIA3 by activating MAPK/ERK signaling pathway

**DOI:** 10.1186/s13045-020-00969-0

**Published:** 2020-10-22

**Authors:** Chun-Hua Wei, Gang Wu, Qian Cai, Xi-Can Gao, Fan Tong, Rui Zhou, Rui-Guang Zhang, Ji-Hua Dong, Yu Hu, Xiao- Rong Dong

**Affiliations:** 1 Cancer Center, Union Hospital, Tongji Medical College, Huazhong University of Science and Technology, 1277 JieFang Avenue, Wuhan, 430022 People’s Republic of China; 2 Experimental Center, Union Hospital, Tongji Medical College, Huazhong University of Science and Technology, 1277, JieFang Avenue, Wuhan, 430022 People’s Republic of China; 3 Institute of Hematology, Union Hospital, Tongji Medical College, Huazhong University of Science and Technology, 1277 JieFang Avenue, Wuhan, 430022 People’s Republic of China

## Retraction Note to: Journal of Hematology & Oncology (2017) 10:125 https://doi.org/10.1186/s13045-017-0493-0

In accordance with the guidelines published by COPE, the authors have retracted this article [1] because of significant concerns regarding a number of Figures presented in this work. In Fig. 3C and 3D there are duplications in the NC and NC-LV groups in the H460 cells, NC-LV, OE-miR-330-3p-LV and anti-miR-330-3p-LV groups in the H1975 cells. In Fig. 7D, the H1975/NC-LV Vec image has been duplicated from the H460/NC image in Fig. 3C. There are multiple duplications throughout Fig. 6, and there are duplications in Fig. 9D (OE-miR-330-3p-LV and OE-miR-330-3p-LV + U0126 groups in the H460 cells, OE-miR-330-3p-LV group in the H1975 cells). The data reported in this article are therefore unreliable.

All authors agree to this retraction.
